# Creation of an intramedullary cavity by hemorrhagic necrosis removal 24 h after spinal cord contusion in rats for eventual intralesional implantation of restorative materials

**DOI:** 10.1371/journal.pone.0176105

**Published:** 2017-04-17

**Authors:** Gabriel Guizar-Sahagun, Angelina Martinez-Cruz, Rebecca E. Franco-Bourland, Eduardo Cruz-García, Alvaro Corona-Juarez, Araceli Diaz-Ruiz, Israel Grijalva, Horacio J. Reyes-Alva, Ignacio Madrazo

**Affiliations:** 1 Research Unit for Neurological Diseases, Instituto Mexicano del Seguro Social, Mexico City, Mexico; 2 Department of Experimental Surgery, Proyecto Camina A.C., Mexico City, Mexico; 3 Department of Biochemistry, Instituto Nacional de Rehabilitación, Mexico City, Mexico; 4 Department of Neurochemistry, Instituto Nacional de Neurología y Neurocirugía, Mexico City, Mexico; 5 Department of Neurology, School of Veterinary Medicine, Universidad Autónoma del Estado de Mexico, Toluca, Mexico; University of Milan-Bicocca, ITALY

## Abstract

Intramedullary hemorrhagic necrosis occurs early after spinal cord injury at the site of injury and adjacent segments. It is considered harmful because of its potential to aggravate secondary injury, and to interfere with axonal regeneration; it might also lead to an unfavorable environment for intralesional implants. Removal of hemorrhagic necrosis has been attempted before with variable results. The invasive nature of these procedures carries the risk of exacerbating damage to the injured cord. The overall objective for this study was to test several strategies for non-damaging removal of hemorrhagic necrosis and characterize the resulting cavity looking for a space for future intralesional therapeutic implants in rats with acute cord injury. Rats were subjected to graded cord contusion, and hemorrhagic necrosis was removed after 24h. Three grades of myelotomy (extensive, medium sized, and small) were tested. Using the small surgical approach to debridement, early and late effects of the intervention were determined by histology and by analytical and behavioral analysis. Appearance and capacity of the resulting cavity were characterized. Satisfactory removal of hemorrhagic necrosis was achieved with all three surgical approaches to debridement. However, bleeding in spared cord tissue was excessive after medium sized and extensive myelotomies but similar to control injured rats after small cord surgery. Small surgical approach to debridement produced no swelling nor acute inflammation changes, nor did it affect long-term spontaneous locomotor recovery, but resulted in modest improvement of myelination in rats subjected to both moderate and severe injuries. Cavity created after intervention was filled with 10 to 15 μL of hydrogel. In conclusion, by small surgical approach to debridement, removal of hemorrhagic necrosis was achieved after acute cord contusion thereby creating intramedullary spaces without further damaging the injured spinal cord. Resulting cavities appear suitable for future intralesional placement of pro-reparative cells or other regenerative biomaterials in a clinically relevant model of spinal cord injury.

## Introduction

Intramedullary hemorrhagic necrosis (IHN) is a pathological process that consistently occurs early after a traumatic spinal cord injury (SCI) [1,2]. It is characterized by the presence of fragments of devitalized cord tissue, cell debris, abundant erythrocytes, and inflammatory cells at the site of injury and adjacent segments.

IHN extension is directly proportional to the severity of the impact [3]. Mechanical forces produced by trauma instantaneously damage neural and vascular structures primarily in the highly vascularized gray matter [4–6]. During the following hours, self-destructive events expand to surrounding gray and white matter, and beyond, to remote sites occupying the central part of the dorsal cord [5–10].

In addition to the damage produced by its mass effect, IHN contributes to secondary damage with toxic substances like heme degradation products or causing oxidative stress and inflammation, among others [6,11–14], and possibly interfering with axonal regeneration [15–17].

Due to its involving in SCI pathophysiology, IHN has for a long-time been a target for therapy. Allen reported a century ago, that a myelotomy (longitudinal midline incision in the spinal cord) together with the removal of “contused tissue” were both structurally and functionally beneficial in injured dogs [18,19] and humans [19]. Since then, only occasional reports of animal studies have shown proof of the benefits from hemorrhagic necrosis removal [20–25]. Reports from uncontrolled clinical trials have suggested both discrete [26,27], and outstanding [28] improvement after IHN removal in spinal cord injured patients. However, removal of IHN has remained an unusual treatment for SCI, possibly due to an unfavorable risk-benefit ratio because it is a highly invasive procedure.

The lack of therapeutic tools for cord healing in humans has encouraged the search for effective reparative interventions. Cell transplantation, and implantation of materials capable of releasing reparative biomolecules to the site of injury have become a major focus of attention in preclinical research as they represent a promising approach to promote neural protection and regeneration, and possibly even lead to the recovery of function after injury [29–31].

Hemorrhagic necrosis after SCI, aside from aggravating secondary injury and interfering with axonal regeneration, is likely to lead to a hostile microenvironment for cells and other reparative materials that could be implanted at the site of lesion, and block the chances for tissue repair [32–34].

Our objective here was to design a safe method for the removal of IHN after moderate or severe spinal cord contusion by debridement that might result in cavities as potential sites for placement of restorative substances or cell transplantation for cord regeneration. To meet this objective, we first tested the risks associated with the extent of three levels of myelotomy lesions as an approach to debridement; the small myelotomy was found to be adequate for IHN removal, with minimal additional risk to contused spinal cords. We further assessed early and late functional outcome of rats subjected to small myelotomies, measured the volumes of their intramedullary cavities by gel injection, and characterized these by histology.

## Material and methods

### Study design

Experiments were conducted in randomized adult female Long-Evans rats, 10 to 12 weeks old, and 240–260 g body weight (n = 166). In a first series of experiments, myelotomies of 3 different lengths were tested as a surgical approach for the removal IHN, and a means to modify the extent of cord swelling after SCI. In later experiments, IHN was removed using only the small surgical approach to debridement (SSAD) in order to: 1) study early effects of SSAD on histological appearance and acute inflammatory reaction after graded cord injury; 2) determine long-term behavioral and histological effects of SSAD after graded cord injury; 3) macroscopically and histologically characterize the appearance of the intramedullary space created after SSAD, as well as to determine its volume by the amount of hydrogel required to fill it. Evaluations were performed by researchers blinded to the experimental groups.

The Ethics Committee in Research of the Instituto Mexicano del Seguro Social approved this study (File number: R-2013-3601-38). All efforts were made to minimize animal pain and discomfort. Experiments were performed in accordance with the National Institute of Health Guide for the Care and Use of Laboratory Animals.

### Anesthesia, injury, cares, and humane endpoint

For cord injury, animals were anesthetized with a mixture of ketamine (80 mg/kg) and xylazine (8 mg/kg) given IM, and for intramedullary surgery, anesthesia was performed using 1.4% inhaled sevoflurane.

Prior to SCI, a laminectomy was performed at T9 under aseptic conditions. After carefully exposing the dural sac without tearing it, rats were suspended in the stereotactic frame clamping them by their spinous processes at T8 and T10. Spinal cord contusion was then produced by using the NYU weight-drop device: onto the exposed dura the 10 g rod was dropped from a height of 25 mm or 50 mm (for moderate or severe intensity injuries, respectively). Errors greater than 3% in height or velocity of impact were not tolerated. Finally, the wound was sutured in layers. Sham-injured animals were not laminectomized. They were only subjected to surgery of soft tissues.

After injury, animals were placed in individual cages with sterile sawdust as bedding, in a clean room, on a 12-h light/dark cycle, and under controlled atmospheric temperature and humidity. Animals were fed with standard rat chow and water *ad libitum*. As prophylactic for infections, 8 mg/kg of ciprofloxacin lactate (Bayer, Mexico City, Mexico) were given subcutaneously every 12 h, starting on the day of surgery and for 7 consecutive days thereafter. To prevent pain and self-mutilation, acetaminophen (Pisa, Mexico) was given at a dose of 30 mg/kg every 12 h, OP, for 1 week. Manual expression of bladders was performed twice a day until bladder function returned.

Euthanasia was performed: 1) at the end of an experiment; 2) in the case of life threatening complications such as pneumonia, severe urosepsis, and abdominal evisceration due to autophagy, and 3) in the case of complications that irreversibly affect the quality of life and interfere with functional evaluations, such as extensive autophagy of hind limbs, exposing muscle and bone. Euthanasia was performed using an overdose of sodium pentobarbital (63 mg) given IP.

### Comparison of myelotomies of 3 different lengths (grades) after severe cord contusion

Three grades of surgical incision (myelotomy) to the injured spinal cord were performed to explore the efficiency of IHN removal, estimate the extent of damage to the injured cord, and modify swelling. Experimental groups were made up as follows: 1) four groups for histology (n = 6, total of 24 animals), one for each of the three grades of myelotomy and one injured control group without myelotomy; 2) five groups for swelling assessment (n = 6, total of 30 rats), the same groups mentioned above, plus one group with sham injury. Rats were subjected to severe cord contusion; after 24 h, myelotomies were performed at the site of injury for removal of IHN. Evaluations were performed 2 days after contusion (1 day after myelotomy or control care).

#### Extensive surgical approach to debridement

The original wound was enlarged enough to expose the dorsal portion of the dura by a laminectomy from T7 to T11. The dural sac from T8 to T10 was cut longitudinally, and then a longitudinal-central myelotomy was performed, involving approximately half of the cord thickness in the central portion of the wound (T9) and one fourth of the cord thickness in adjacent segments (T8 and T10). For myelotomy, dissections were performed initially with sharp microsurgery instruments and then blunt dissections aiming to preserve the integrity of great vessels. The inside of the exposed cord was then gently irrigated with artificial cerebrospinal fluid (prepared as recommended by Alzet: http://www.alzet.com/products/guide_to_use/cfs_preparation.html). The dural sac was repaired by placing a fragment of autologous fascia (obtained from paraspinal-exposed muscles) on the open dura, which was sealed at the periphery with fibrin glue. Finally, the surgical incision was sutured in layers.

#### Medium sized surgical approach to debridement

Basically the same procedure was performed here as that described above for the extensive myelotomy surgery, with minor changes: laminectomy was only extended from T8 to T10; myelotomy was about 3-mm-long at T9, involving approximately half of the cord thickness. Intramedullary irrigation, dura repair, and surgical wound closure were performed as previously described.

#### Small surgical approach to debridement (SSAD)

The area of the injured cord, showing the greatest damage was identified, judging from the presence of a subarachnoid hematoma, as well as from an intense dark brown/purple color on the cord surface. Here a longitudinal 2-mm-long incision of the dural sac was performed to allow for subarachnoid hematoma drainage. It was not necessary to extend the laminectomy. The exposed cord was then punctured right at the site of the greatest damage (usually dorso-lateral) using a 33-gauge needle with a blunt point (style 3) affixed to a Neuros™ syringe (Model 1705 RN, Hamilton Co., Reno NV). To ensure penetration depth, the sleeve of the needle of the Neuros system was placed 1.5 mm from the tip of the needle; the tip was then lowered on to the surface of the cord and from there further lowered 1.5 mm into the tissue. Then, a 1-mm-long myelotomy was made by moving the needle back and forth cranio-caudally. Just by removing the needle, a portion of IHN came out spontaneously. Then, to complete removal of necrotic tissue, especially of large fragments of devitalized material, the intramedullary area had to be aspirated and irrigated, as follows: a polyethylene tubing (PE10, Intramedic, Becton Dickinson Co, Sparks MD) attached to an ultra-fine insulin syringe was introduced through the myelotomy and hematic material was gently aspirated. Then, using tubing of the same caliber, the exposed intramedullary region was further gently irrigated using artificial cerebrospinal fluid until it returned colorless (on average, 1 mL). Finally, one last aspiration was performed as described above. At this time, the presence of an intramedullary cavity was corroborated by vision through the surgical microscope. The procedure was finalized by suturing the dural sac with one stitch using 10–0 nylon (fascia and fibrin glue were no necessary for dural repair); the surgical wound was sutured in layers.

#### Control surgical approach

Twenty-four hours after injury, rats were anesthetized, the original surgical wound was reopened, and finally sutured. The spine and cord were not manipulated at all.

#### Methods to evaluate histology and swelling

For histology, rats were given 1,000 U of heparin IP and were deeply anesthetized. They were fixed by intra-cardiac puncture. Initially they were perfused with 200 mL saline, followed by 500 mL 10% buffered formaldehyde.

A 10-mm-long segment of cord centered at the site of injury was removed, post-fixed for 1 week in the same fixative, and processed for paraffin embedding. Six-micron thick serial transverse sections were cut at 1500 μm intervals. They were stained with hematoxylin/eosin and Masson trichrome standard methods. The section with the least amount of spared cord tissue was defined as the lesion epicenter. A qualitative and quantitative (morphometric) estimate of both IHN and bleeding in spared cord tissue were performed at the lesion epicenter, as well as 1.5 mm and 3.0 mm cranial and caudal to it by an expert SCI morphologist.

For morphometric analyses, digital images of transverse sections of cords stained using the Masson technique, were captured with a camera (Evolution MP color from Media Cybernetics, V 6.1, Silver Spring, MD) affixed to a bright-field microscope (Olympus BX51 TRF from Olympus Corporation, Tokyo Japan). Measurements were carried out using the Image-Pro Premier image analysis software from Media Cybernetics, V 9.0.

To measure the amount of IHN, that is “surgically removable unwanted material”, the "region of interest" (ROI) was defined as the bloody fragmented amorphous spinal cord tissue and debris observed at the site of lesion and was traced manually using the polygon tool accompanying the software. With the automatic dark objects threshold tool of the software, all dense material within this space, was automatically selected and quantified. Results are reported in mm^2^.

Bleeding was quantified by measuring the bloody area within the spared cord tissue, as follows: after manual tracing of ROI (areas of spared cord tissue), the intense black-red color of the erythrocytes, was selected manually using the toggle threshold dialog of the software, carefully avoiding non-specific background. For color selection on the image, a 1X1 pixel size was utilized, with HIS (hue saturation intensity) in the image interpretation mode. Total bleeding area for each section was obtained by the sum of all dotted areas. Results (average of 3 measurements) are reported in mm^2^.

To assess cord swelling, animals were deeply anesthetized and euthanized. Immediately thereafter, a 15 mm-long segment of spinal cord centered at the site of injury was removed, weighed (wet weight), dried at 56°C for 6 h and reweighed (dry weight). Swelling is presented as percentage of water in cord specimens, that is, % water content = [(wet weight—dry weight)/wet weight] x 100.

### Assessment of early effects of SSAD after graded cord contusions

Morphologic assessment was made to cord tissue of moderately and severely contused rats 1 h and 24 h after SSAD or control surgery (eight experimental groups, n = 4, total of 32 animals). Euthanasia, as well as tissue processing and staining were carried out as described in the previous section, except that the spinal cord was sectioned in the sagittal plane.

Acute inflammatory reaction was estimated in rats subjected to severe injury, by myeloperoxidase (MPO) activity as indirect marker for neutrophil infiltration. Three experimental groups were used (n = 8, total of 24 animals): injury plus debridement, injured control, and sham injury. Rats were decapitated 24 h after SSAD or control surgery and 15-mm-long cord tissues from the sites of injury, including meninges, were removed, weighed, frozen on dry ice, and stored at − 80°C.

MPO activity was measured as previously described [35]. Briefly, tissues were homogenized in ice-cold phosphate buffer (PB). Samples were centrifuged at 30,000xg. Pellets were resuspended in PB, centrifuged as described above, and resuspended in 0.5% hexadecyltrimethylammonium bromide in PB, subjected to three freeze-thaw cycles, and finally ultrasonic disruption. After 20 min incubation at 48°C, samples were centrifuged at 12,000xg; supernatants were collected for MPO activity. Reaction was initiated by mixing 0.1 mL aliquots of the sample with 2.9 mL of PB containing 0.167 mg/mL of o-dianisidine dihydrochloride and 0.0005% H_2_O_2_. Absorbance at 460 nm was recorded for 2 min every 15-sec in a UV-visible spectrophotometer (Lambda 20; Perkin Elmer). One international unit (IU) of MPO activity was defined as 1 μmol H_2_O_2_ metabolized per min at 25°C. Results were expressed as IU of MPO per gram wet tissue.

### Assessment of long-term effects of SSAD after graded cord injury

Rats with moderate or severe intensity injuries subjected to SSAD and controls (four experimental groups, n = 10, total of 40 rats) were initially used for evaluating locomotor performance, as well as general health and wellbeing; then, rats that survived 8 weeks after injury were euthanized for morphologic analysis.

#### Assessment of locomotor performance

Contused rats were assessed individually for 3 min in an open field using the Basso, Beattie, and Bresnahan (BBB) rating scale [36]. Evaluations were performed weekly for 8 weeks, by two experienced observers; the final score was the average of both observers’ assessments.

#### Assessment of general health and wellbeing

As an indicator of general health and wellbeing after injury, body weight was monitored weekly for 8 weeks. Deaths were registered as an index of animal survival rate.

#### Long-term morphologic analysis

Morphologic studies were performed in specimens from rats subjected to moderate (n = 9) and severe (n = 7) injury, subjected to SSAD or control surgery. Euthanasia, tissue processing, and morphometry were performed as described above.

Amount of spared cord tissue in transverse sections of cords stained using the Masson technique at the injury epicenter, as well as 1.5 mm and 3.0 mm cranial and caudal to it, were estimated by automatic quantification of ROI traced manually using the polygon tool of the software. Results are reported in mm^2^.

To precisely identify myelination, immunostaining against myelin basic protein (MBP) was performed in sections from the epicenter and 1.5 mm cranial and caudal to it. Sections were deparaffinized, rehydrated, and subjected to antigen retrieval (using citrate buffer and microwave heating). Endogenous peroxidase was blocked with 3% hydrogen peroxide in deionized water; thereafter sections were incubated in 5% horse serum in PBS to decrease nonspecific staining. Sections were incubated in mouse monoclonal anti-MBP (1:600, SC-71546, Santa Cruz Biotechnology, Inc.) for 30 min at RT. The primary antibody was detected using the MACH 4 Universal HRP-Polymer Kit (M4U534, Biocare Medical) according to manufacturer’s instructions, and were developed with DAB (Betazoid DAB Chromogen Kit, BDB2004, Biocare Medical). Finally, sections were counterstained with hematoxylin. Negative controls were obtained by omitting the primary antibody from the immunostaining procedure.

Myelination was quantified by measuring (in triplicate) the area immunostained for MBP within the ROI (spared cord tissue). Brown colored immunostaining was manually selected using the toggle threshold dialog of the software, carefully avoiding nonspecific background. For each section, a mask image was created with thousands of dots; total immunostained area for each section was obtained by the sum of all dotted areas. Results (average of 3 measurements) are reported in μm^2^.

### Description of the intramedullary space after IHN removal

By measuring the number of microliters (volume) of a generic hydrogel injected with a Hamilton syringe into the intramedullary space resulting after IHN removal, and before gel started to overflowed, it was possible to adequately determine the size of the space in rats with both moderate and severe intensity contusions (two experimental groups, n = 8, total of 16 rats). The same rats were euthanized at 1 h and 24 h post-intervention for macroscopic (n = 4) and histologic (n = 4) observation of the site of injury.

For macroscopic observations, rats were deeply anesthetized and perfused to remove intravascular blood, as previously described. Cords were dissected out, sectioned transversally at the site of injury, and the cross-sectional area was photographed using a digital camera (Sony Cyber-Shot HX50V).

For histology, euthanasia and tissue processing were performed as described above. Six-micron thick transverse sections from epicenter were stained with hematoxylin/eosin.

### Statistical analysis

Statistical analyses were performed using Prism V6.0e for Mac OS X. Areas of IHN and bleeding in acute injuries were analyzed by Kruskal-Wallis test followed by Dunn's multiple comparisons test. Areas of spared cord tissue and myelination were analyzed by Kolmogorov-Smirnov test and Student’s t-test, respectively. Cord swelling and MPO activity were analyzed by one-way ANOVA followed by Bonferroni’s tests. Locomotor function (BBB) data and changes in body weight were analyzed using the repeated measures ANOVA test. Animal survival rate was analyzed with the Log-rank test and presented as Kaplan-Meier survival plots. Differences were considered significant when *p* < 0.05.

## Results

### Removal of IHN from severely contused spinal cords: Effects of extent of graded myelotomies

**Fig 1:**
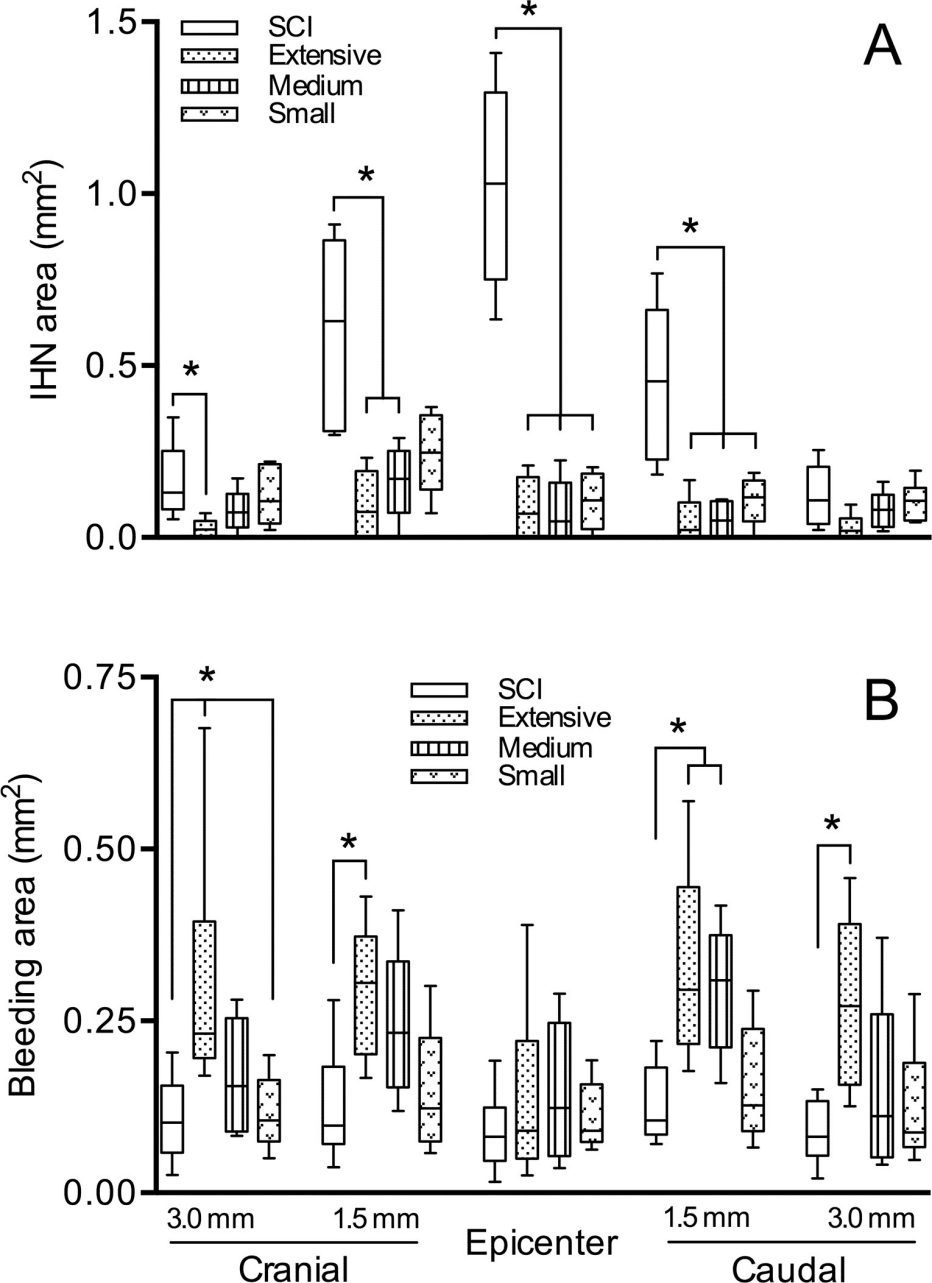
Effects of extent of myelotomies on removal of intramedullary hemorrhagic necrosis (IHN) and bleeding in spared cord tissue. Plots show measurements (in mm^2^) of IHN (A) and bleeding in spared spinal cord (B) in transverse cord sections at the epicenter, as well as 1.5 mm and 3.0 mm cranial and caudal to it. Spinal cords were harvested 48 h after severe contusion/24 h after debridement in the following groups of rats: untreated cord injured (SCI), and cord injured plus extensive, medium or small surgical approaches. Data are expressed as boxes and whiskers (n = 6). Boxes extend from the 25th to 75th percentiles; the line inside the box is the median; whiskers go from the lowest to the largest values in that group. Statistical analysis: Kruskal-Wallis test was performed separately for the four groups at each cord level, and was followed by Dunn's multiple comparisons test. *, *p* < 0.05.

As depicted in Fig 1A, morphometric analyses revealed that the greatest amount of IHN removed from the epicenter, as well as from segments 1.5 mm cranial and caudal to it, was from severely contused rats subjected to extensive and medium sized surgical debridement approaches, albeit at the cost of severe bleeding in the spared cord tissue, as shown in Fig 1B, particularly in the case of extensive myelotomies.

As seen in Fig 1A, in the case rats subjected to SSAD, removal of IHN at the epicenter and 1.5 mm caudal to it was almost equivalent to that achieved using extensive and medium sized myelotomies, although slightly less in cranial sections, where the remaining amount of IHN was similar to that seen in corresponding control tissue. As illustrated in Fig 1B, bleeding observed in spared cord tissue of SSAD treated rats, was as small as that seen in controls.

**Fig 2:**
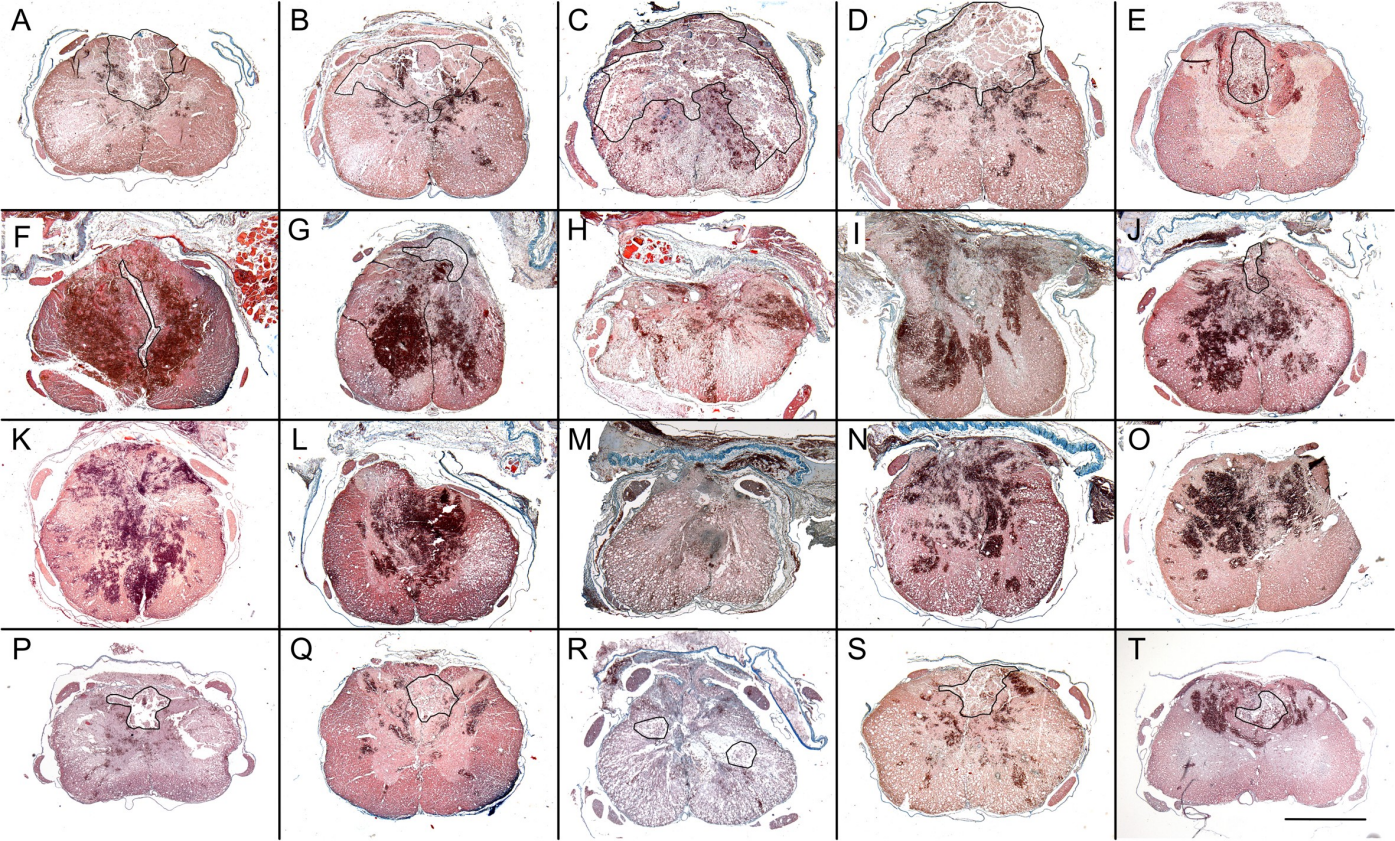
Histologic appearance of intramedullary hemorrhagic necrosis (IHN) and cord bleeding after graded myelotomies. Representative transverse spinal cord images (obtained 48 h after severe contusion/24 h after debridement) of injured-untreated controls (A-E), as well as of extensive (F-J), medium (K-O), and small (P-T) surgical approaches. Images correspond to the epicenter (C, H, M, and R), 3.0 mm (A, F, K and P) and 1.5 mm (B, G, L and Q) cranial to it, as well as 1.5 mm (D, I, N, and S) and 3.0 mm (E, J, O, and T) caudal to it. IHN area is outlined. The intense black-red color reveals bleeding in spared cord tissue. Masson's stain. Scale bar = 1 mm.

Shown in Fig 2 are representative histologic images of IHN and extent of spinal cord bleeding in spared tissue sections from the site of lesion (epicenter), and sections 1.5 and 3.0 mm cranial and caudal to it, of severely contused rats subjected to graded myelotomies.

**Fig 3:**
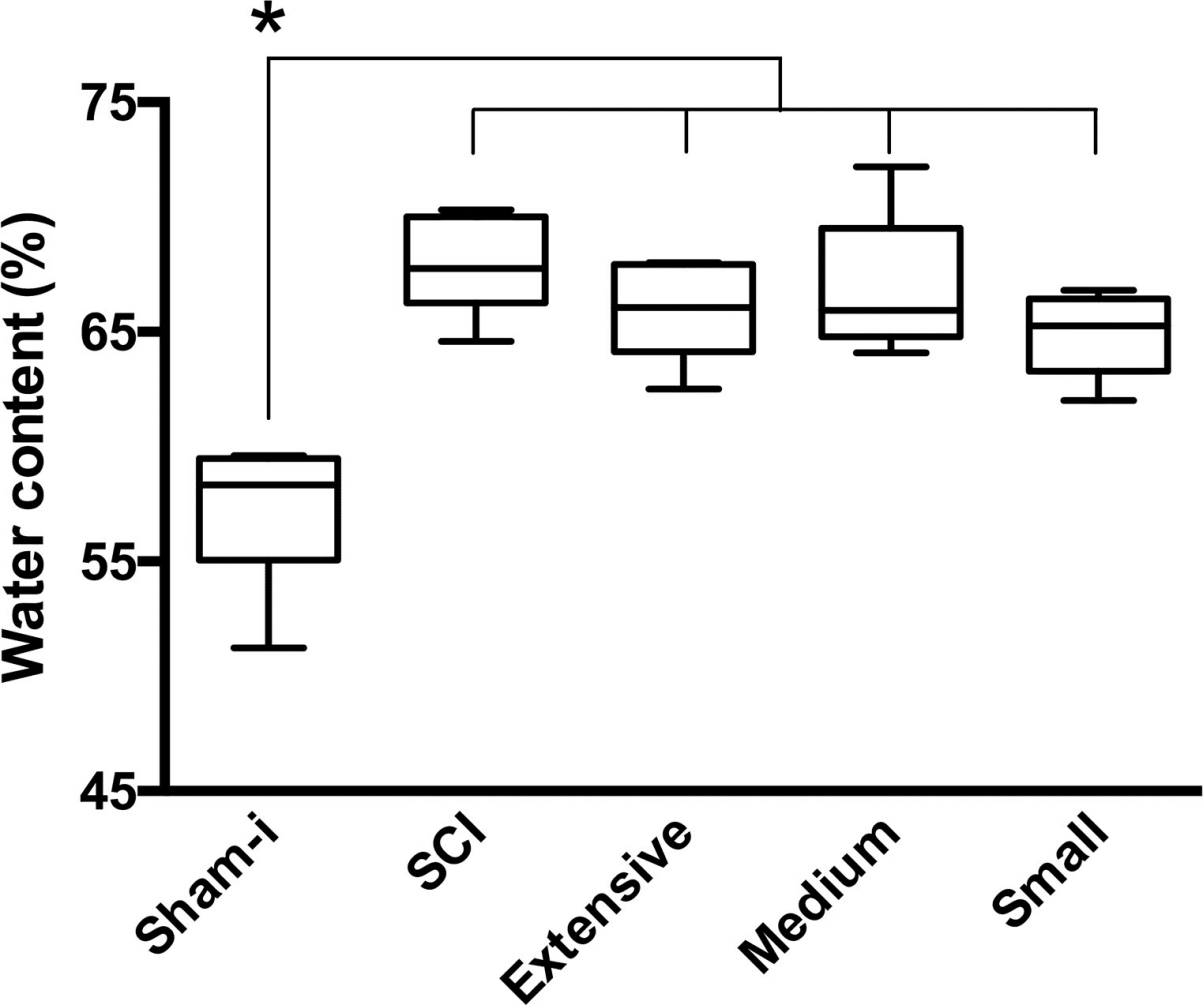
Effects of extent of myelotomies on cord swelling. Plots show percentage of water content in the spinal cords 48 h after contusion/24 h after debridement in the following groups of rats: sham-injured (Sham-I), untreated cord injured (SCI), and cord injured plus extensive, medium or small approaches. Data are expressed as box and whiskers (n = 6). The box extends from the 25th to 75th percentiles; the line inside the box is the median; whiskers go from the lowest to the largest values in that group. Statistical analysis: one-way ANOVA followed by Bonferroni’s test, * *p* < 0.05.

Percentage of water content in cords from all groups of injured rats, was significantly increased (average 9.2%, *p <* 0.05) compared with rats with sham-injury. No significant differences were found among injured rats regardless of the extent of the intervention (Fig 3).

### Early effects of SSAD after graded cord injury

**Fig 4:**
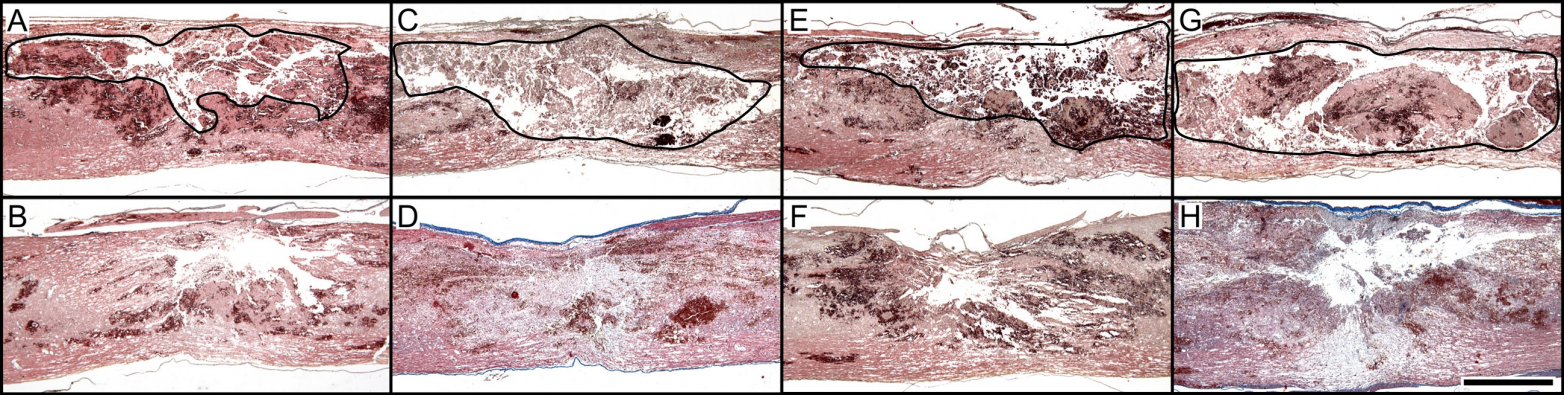
Histologic appearance of early effects of removal intramedullary hemorrhagic necrosis (IHN) after graded cord injury by small surgical approach to debridement (SSAD). Representative sagittal spinal cord images of moderate (A-D) and severe (E-H) injuries, untreated (A, C, E, and G) or subjected to SSAD (B, D, F, and H). Images correspond to rats euthanized 1 h (A, B, E, and F) and 24 h (C, D, G, and H) after intervention or control manipulation. Area of IHN in control rats is outlined. Masson’s stain. Scale bar = 1 mm.

**Fig 5:**
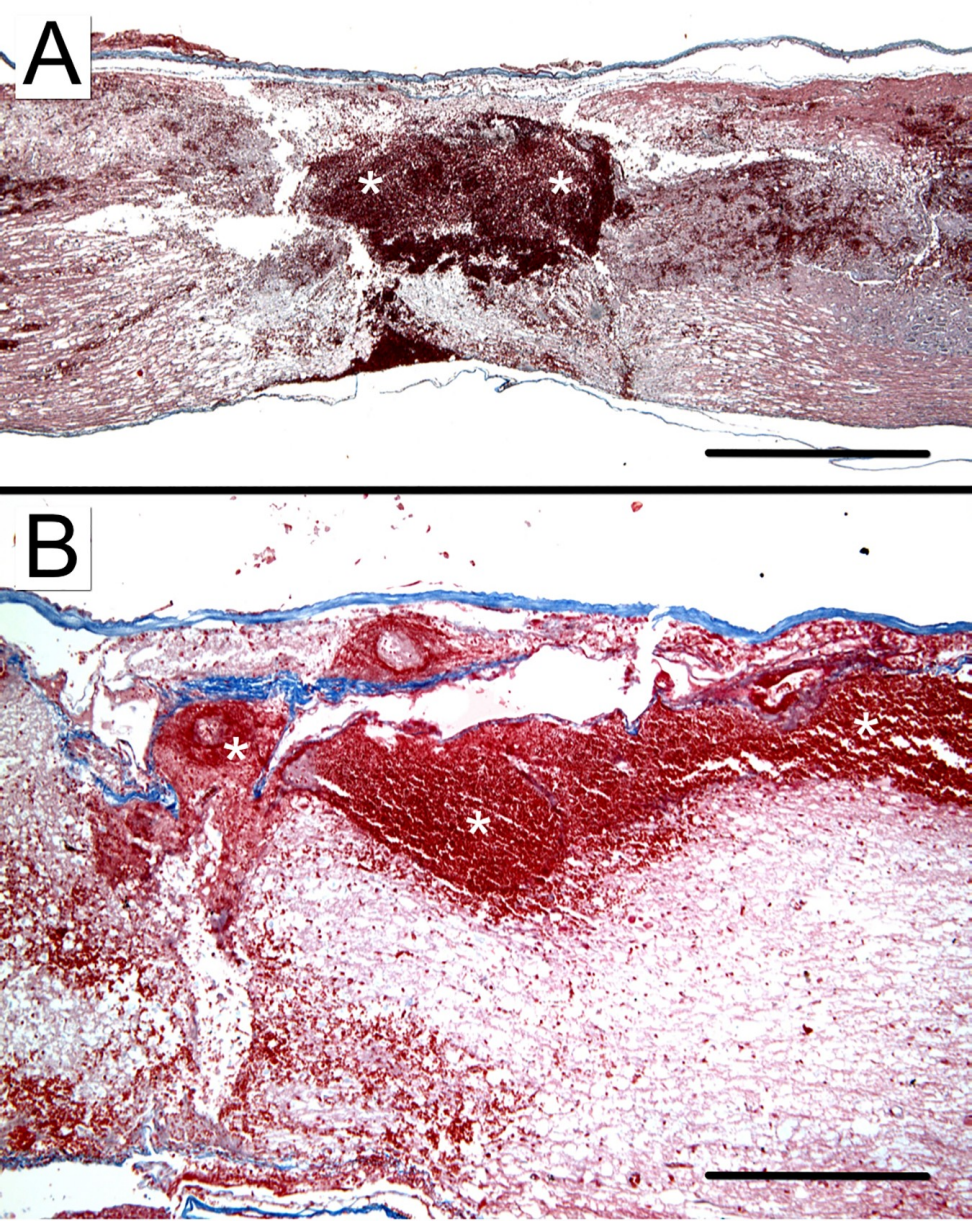
Histologic appearance of complications attributable to small surgical approach to debridement (SSAD). Sagittal images show undesirable intramedullary (A) and subarachnoid (B) extensive bleeding (asterisks) in specimens of injured rats subjected to SSAD 24 h (A) and 1 h (B) before euthanasia. Masson’s stain. Scale bars: A, 1 mm; B, 400 μm.

In injuries of both moderate and severe intensity, SSAD allowed removal of IHN which resulted in empty spaces containing only scarce debris, as demonstrated at 1 h and 24 h after intervention (Fig 4). Usually results were satisfactory, but unwanted extensive intramedullary and subarachnoid bleeding secondary to the intervention did occur in 2/16 cases (12.5%, Fig 5).

**Fig 6:**
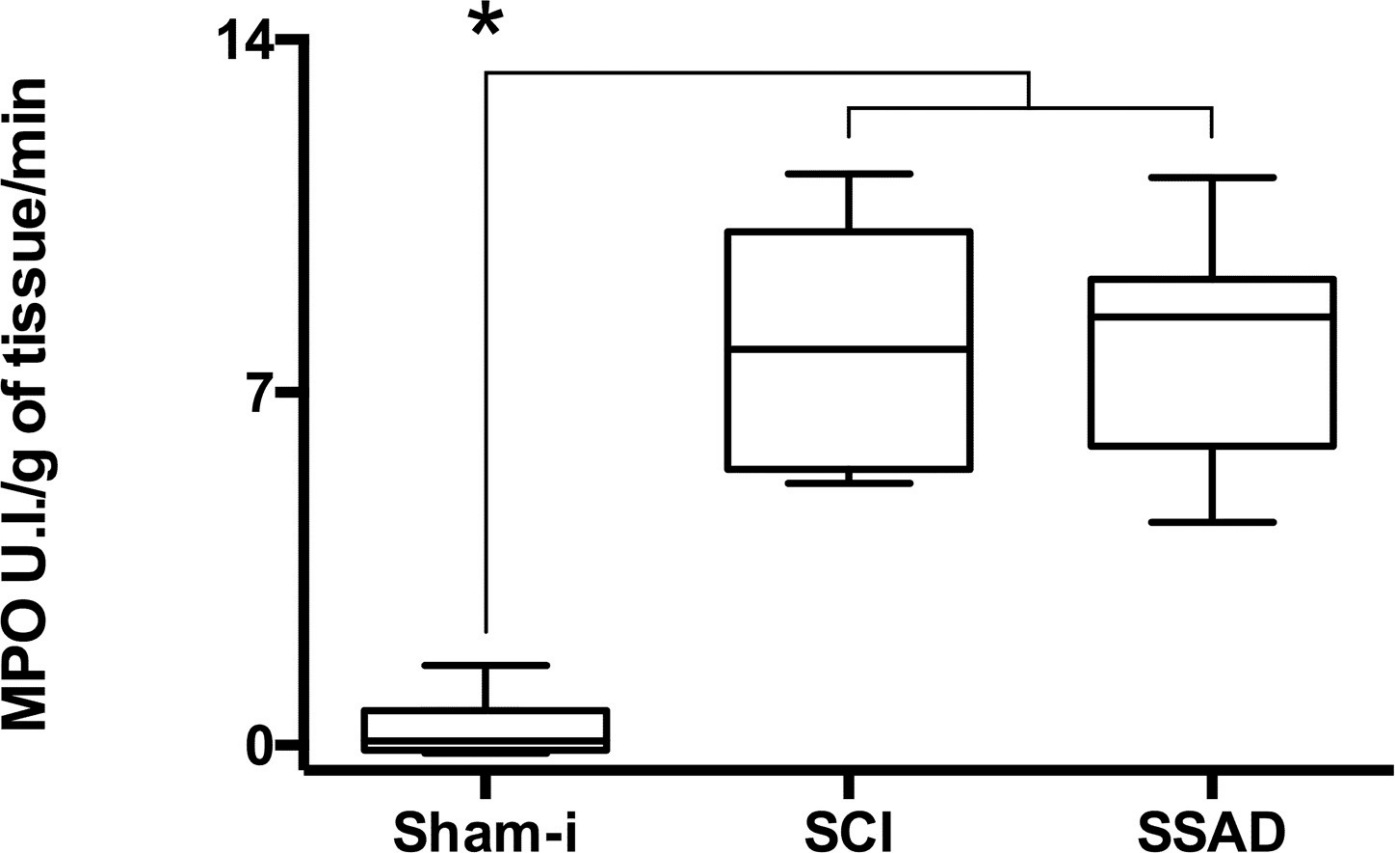
Effects of small surgical approach to debridement (SSAD) on acute cord inflammation. Plots show myeloperoxidase (MPO) activity in rats at 48 h after severe cord injury/24 h after intervention. Groups are as follows: sham-injury (Sham-I), untreated injured rats (SCI) and rats subjected to SSAD. Data are expressed as box and whiskers (n = 8). Statistical analysis: one-way ANOVA followed by Bonferroni’s test, * *p* < 0.05.

MPO activity, used here as an indirect marker for neutrophil infiltration, increased significantly at 48 h after severe cord contusion in both rats subjected to SSAD and untreated SCI controls, in comparison with sham-injured rats (*p* < 0.05). The intervention did not affect MPO activity which was almost the same for both groups of injured rats (Fig 6).

### Long-term effects of SSAD after graded cord injury

#### Motor function outcome

**Fig 7:**
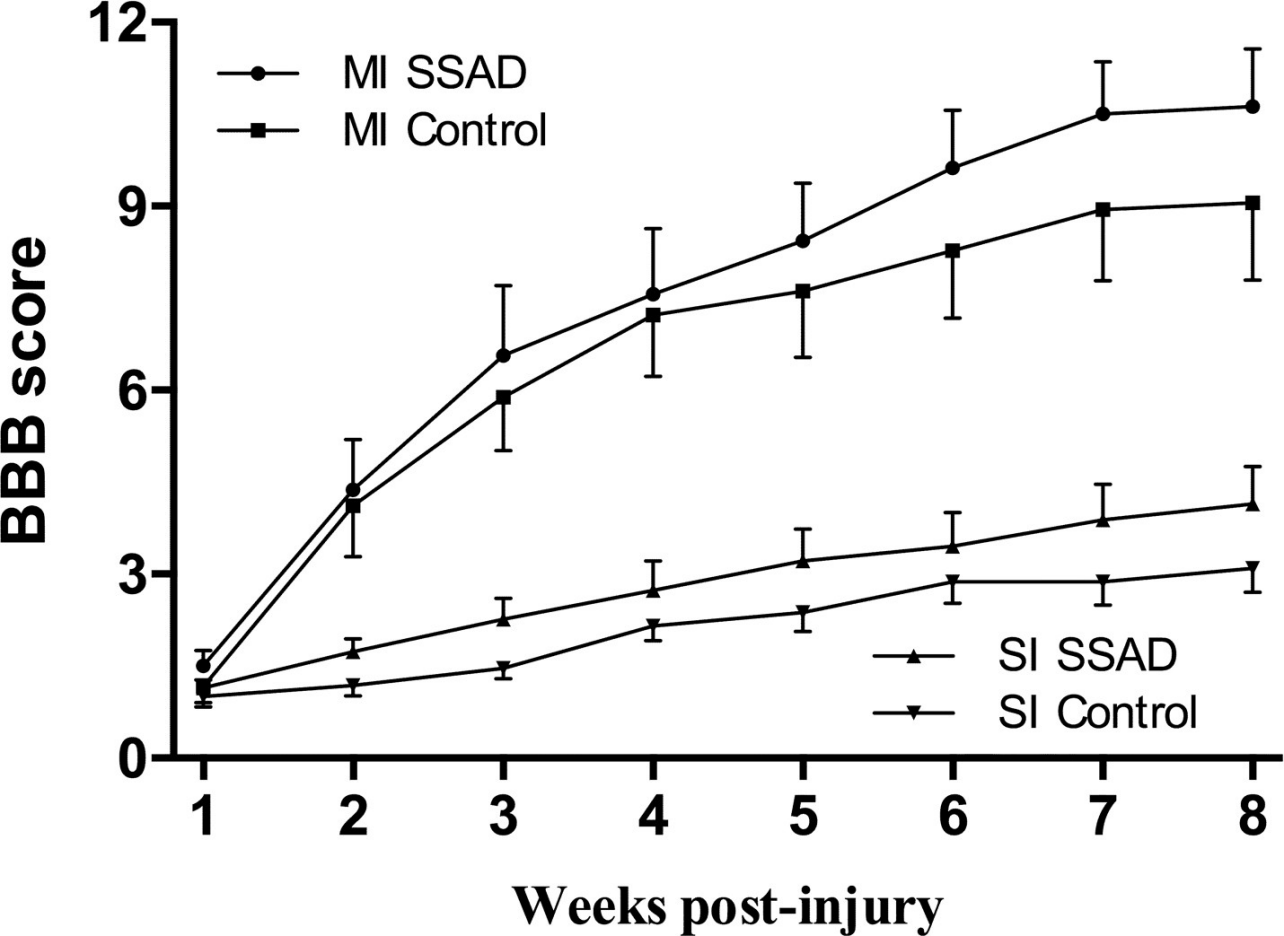
Effect of small surgical approach to debridement (SSAD) on hind limb motor function scores (Basso-Beattie-Bresnahan scale). Plots show locomotor performance over a period of 8 weeks after moderate (MI) and severe (SI) intensity injuries to the spinal cord. Data are expressed as the mean ± S.E.M. (n = 10). Statistical analysis: Repeated measures ANOVA was performed separately for SSAD vs. control MI and SI scores; no significant differences were found.

All animals, regardless of severity of injury and treatment, showed flaccid bilateral hind limb paralysis at day 1. Hind limb performance gradually improved thereafter until the end of the study. At week 8 after injury, the average BBB score for rats subjected to injury of moderate intensity was 10.63 and 9.06 in the SSAD and control groups, respectively. In rats subjected to injury of severe intensity, the final average BBB score was 4.14 and 3.09 in the SSAD and control groups, respectively. Differences were statistically non-significant comparing intervention with controls in both moderate and severe injuries (Fig 7).

#### General health and wellbeing

**Fig 8:**
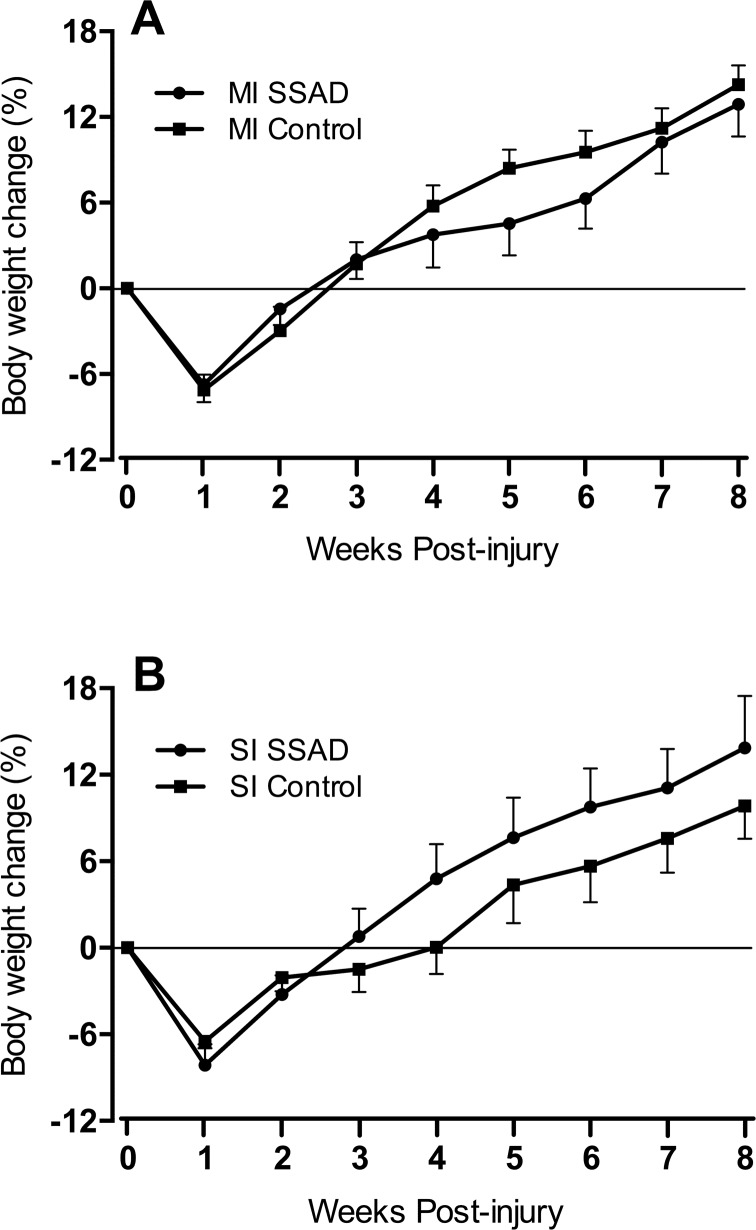
Effect of small surgical approach to debridement (SSAD) on body weight of contused rats. Percent body weight changes over the course of 8 weeks post-SCI. A, rats subjected to injury of moderate intensity (MI) and SSAD and untreated controls. B, similar conditions for severely injured rats (SI). Data are expressed as the mean ± S.E.M. (n = 10). Statistical analysis: Repeated measures ANOVA was performed separately for SSAD vs. control MI and SI scores; no significant differences were found.

All rats lost body weight the first week post-injury regardless of severity of injury and treatment; then the trend was reverted and rats gained weight progressively for the remainder of the study. In rats with a moderate SCI and SSAD, average weight ranged between -6.7% and 12.9% (from their pre-contusion weight), and between -7.1% and 14.3% in untreated controls (Fig 8A). For severely injured rats, body weight ranged between -8.1% and 13.9% in the treated rats, and between -6.5% and 9.8% in the untreated controls (Fig 8B). Differences were not statistically significant. For reference, after 8 weeks, sham-injured rats gained an average of 25.8% of their baseline weight.

**Fig 9:**
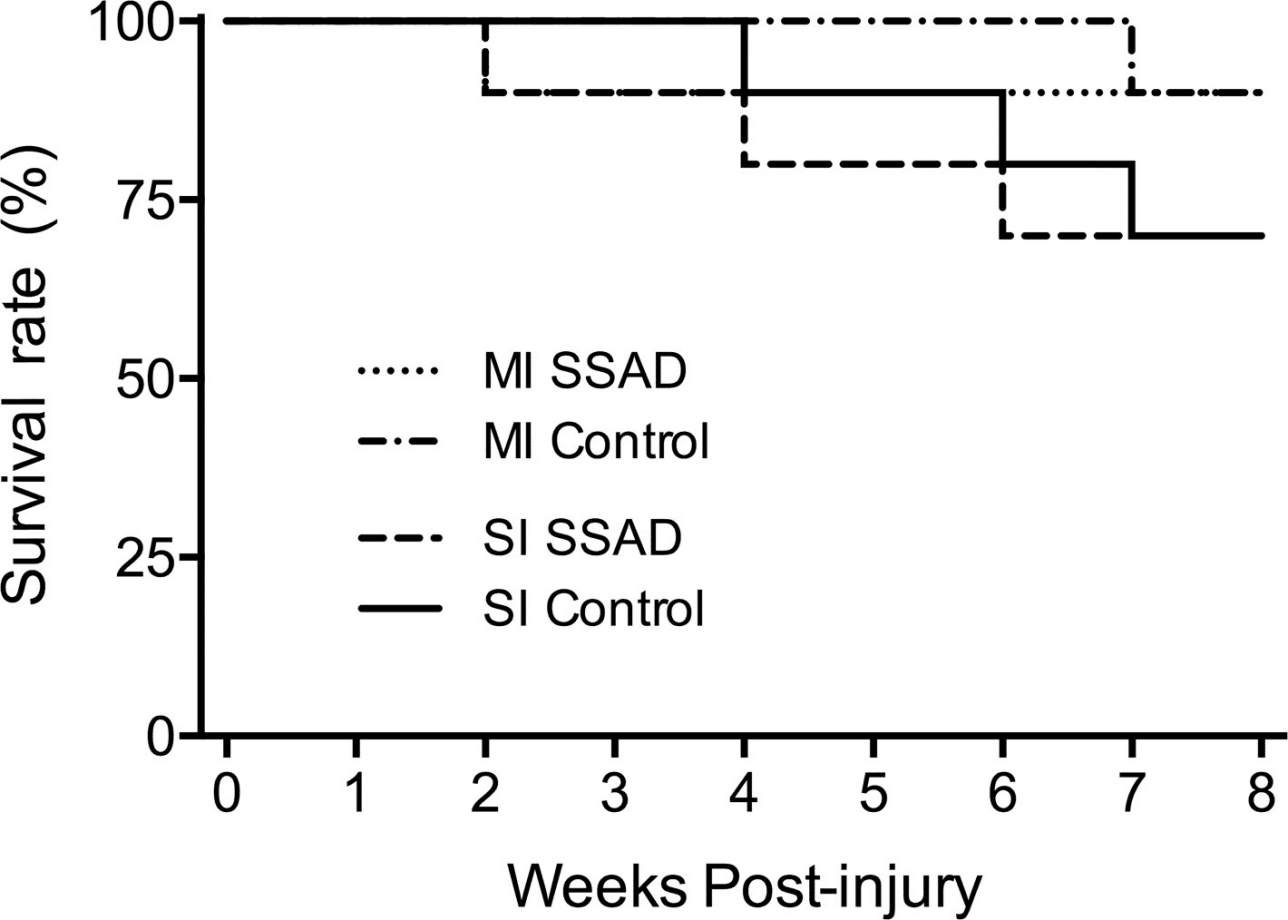
Effect of small surgical approach to debridement (SSAD) on long-term survival rate. Kaplan-Meier survival plots show no differences between debrided and untreated control rats subjected to injuries of moderate (MI) or severe (SI) intensities. Statistical analysis: Log-rank test.

SSAD had no effect on rats’ long-term survival rate. For rats subjected to injury of moderate intensity, survival rate was 90% (9/10): one rat intervened by SSAD was euthanized 2 weeks post-injury because of abdominal evisceration due to autophagy, and one control rat was found dead seven weeks post-injury, for no apparent cause. For rats with severe injuries, survival rate was 70% (7/10): for treated rats, two were sacrificed on account of pneumonia and one because severe urosepsis; in the case of untreated controls, one was sacrificed because of pneumonia and two for extensive autophagy of hind limbs (Fig 9).

#### Long-term morphologic outcome

**Fig 10:**
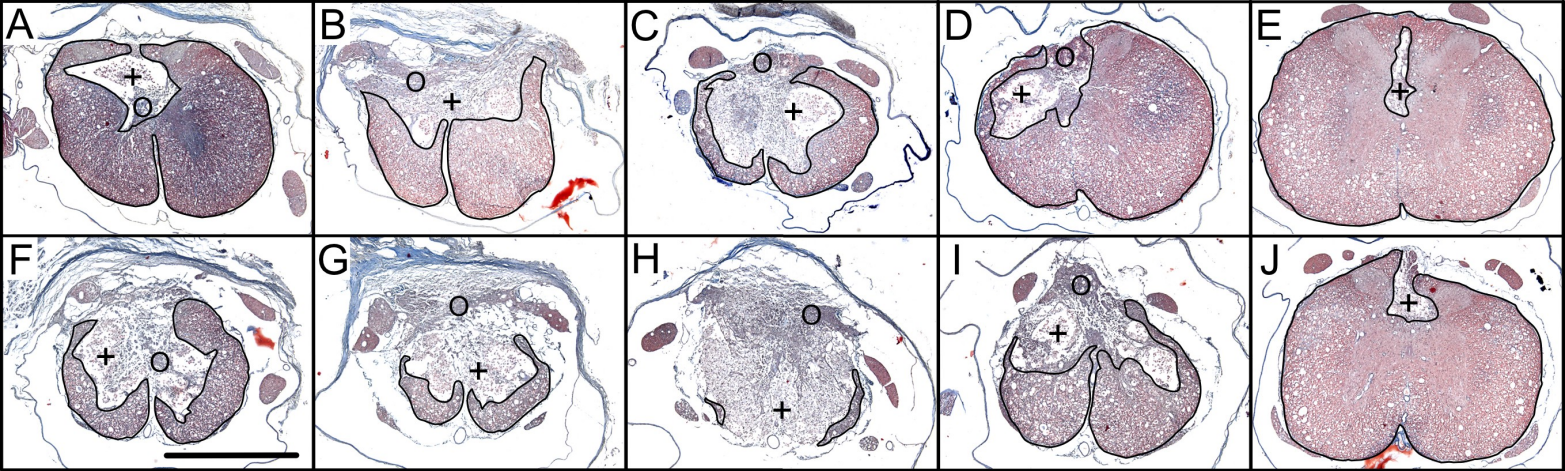
Histologic appearance of contused spinal cord 8 weeks post-injury. Representative transverse images of the contused cord of a rat subjected to an injury of moderate intensity (A-E), and images of a rat subjected to severe injury (F-J). Sections correspond to the epicenter (C, H), as well as to adjacent segments: 1.5 mm (B, G) and 3 mm (A, F) cranial to it, and 1.5 mm (D, I) and 3 mm (E, J) caudal to it. The spared cord tissue is outlined. +, cavities containing abundance of macrophages; O, area of newly formed tissue. Masson’s stain. Scale bar = 1 mm.

At 8 weeks post-injury, rats subjected to SSAD and untreated controls showed similar general histologic characteristics when contrasted separately based on their injury severity and cord location in relation to the site of lesion at the epicenter. Usually, specimens of the epicenter showed a variable amount of spared white matter mainly located at ventral and lateral margins; a major portion of the epicenter was occupied by cavitation(s) containing macrophages and newly formed tissue. As sections move away from epicenter, more spared white and gray matters were observed; likewise, the cavity size was progressively smaller, in both cranial and caudal directions (Fig 10).

**Fig 11:**
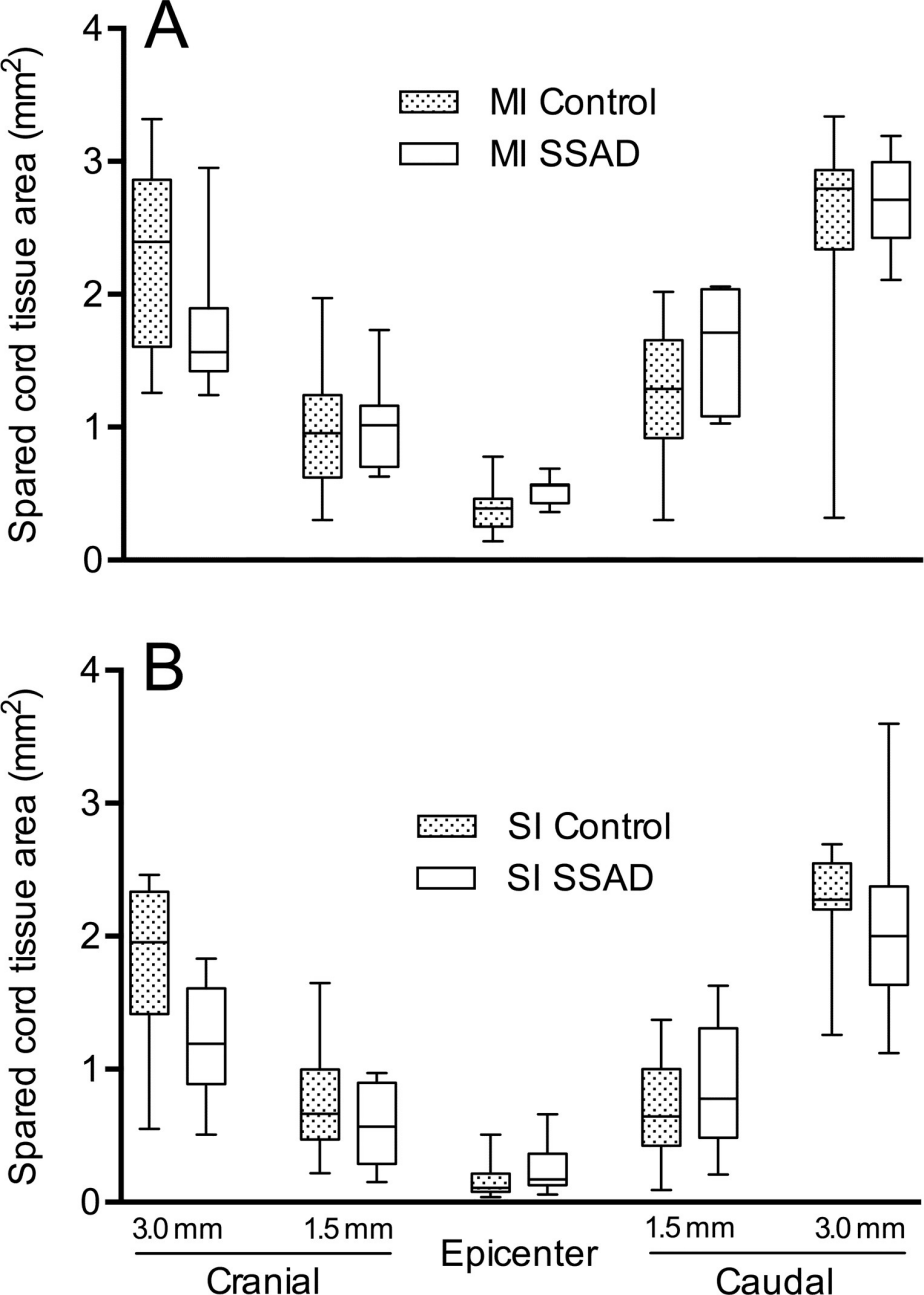
Area of spared cord tissue 8 weeks after injury. Box and whiskers graphs depict morphometric data (mm^2^) obtained from transverse cord sections at the epicenter, as well as 1.5 mm and 3.0 mm cranial and caudal to it. A, rats subjected to injury of moderate intensity (MI, n = 9). B, animals injured with severe intensity (SI, n = 7). SSAD, small surgical approach to debridement. Statistical analysis: Kolmogorov-Smirnov test was performed separately for each level of injury severity (intensity), and cord level. *, *p* < 0.05.

Differences between the amount of spared cord tissue at the epicenter and adjacent cranial and caudal segments as a function of injury intensity and SSAD were not statistically significant (Fig 11).

**Fig 12:**
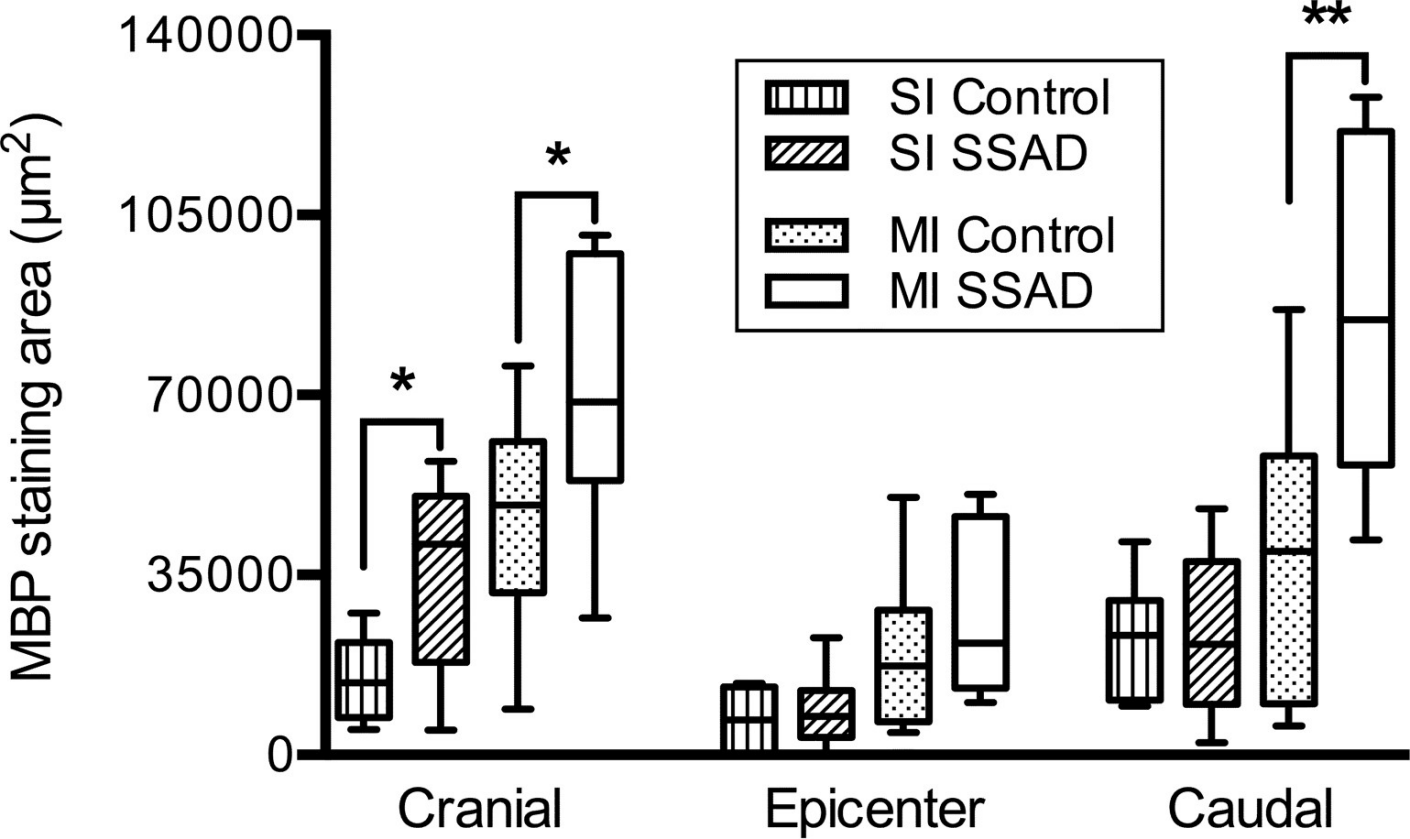
Area of myelination of spared spinal cord tissue 8 weeks after injury. Box and whiskers graphs showing measurements (in μm^2^) of MBP immunostaining in transverse cord sections at the epicenter, and 1.5 mm cranial and caudal to it. SI, injuries of severe intensity (n = 7); MI, injuries of moderate intensity (n = 9); SSAD, small surgical approach to debridement. Statistical analysis: Student’s t test was performed separately at each cord level, for each injury intensity. *, *p* = 0.03; **, *p* = 0.009.

**Fig 13:**
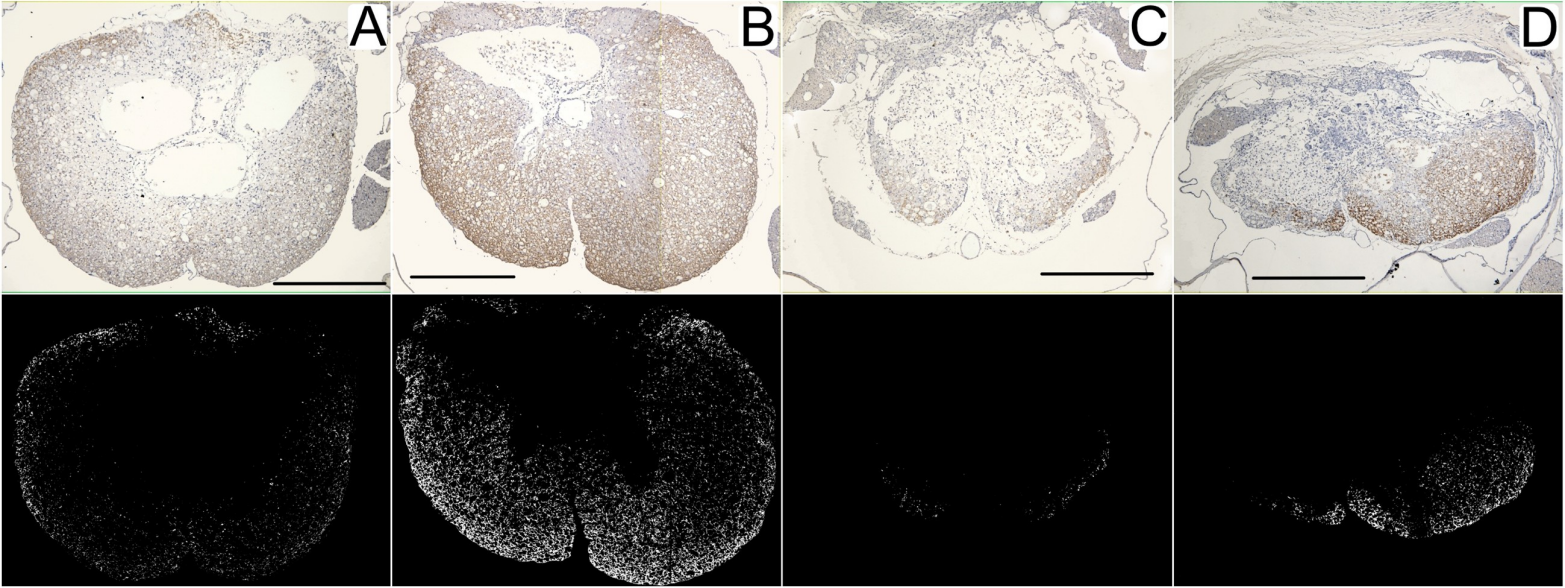
Histologic appearance of myelination 8 weeks post-injury. Representative transverse image of MBP immunostaining of rats subjected to injuries of moderate (A, B) and severe (C, D) intensity; A and C, were controls and B and D were rats subjected to a small surgical approach to debridement. Images correspond to sections obtained 1.5 mm cranial to the epicenter. Scale bar = 0.5 mm. Histological images (above) are shown with their corresponding masks (below); immunostained marks are seen in areas of spared tissue.

Results of morphometric analysis of MBP immunostaining, used to estimate the grade of myelination in spared cord tissues, are shown in Fig 12. It can be seen there are no differences in the amount of myelin at the epicenter between rats that underwent SSAD and controls, for both moderate and severe SCIs. Cranially, 1.5 mm from the epicenter, rats subjected to SSAD show increased myelination compared to controls for both severe and moderate injuries (*p* = 0.03). Caudally, 1.5 mm from the epicenter, rats subjected to SSAD show greater myelination compared to controls for moderate injuries (*p* = 0.009); there were no significant differences between severe injuries and controls. Representative images of MBP immunostaining and masks created from them are shown in Fig 13.

### Characteristics of the space created by removal of intramedullary hemorrhagic necrosis

Volume of hydrogel used to fill intramedullary spaces created after removal of IHN by SSAD, just before it started to overflowed, went from 10 to 15 μL: 11.6 ± 1.8 SD in rats subjected to moderate injury, and 12.6 ± 1.9 SD, after severe contusion (n = 6).

**Fig 14:**
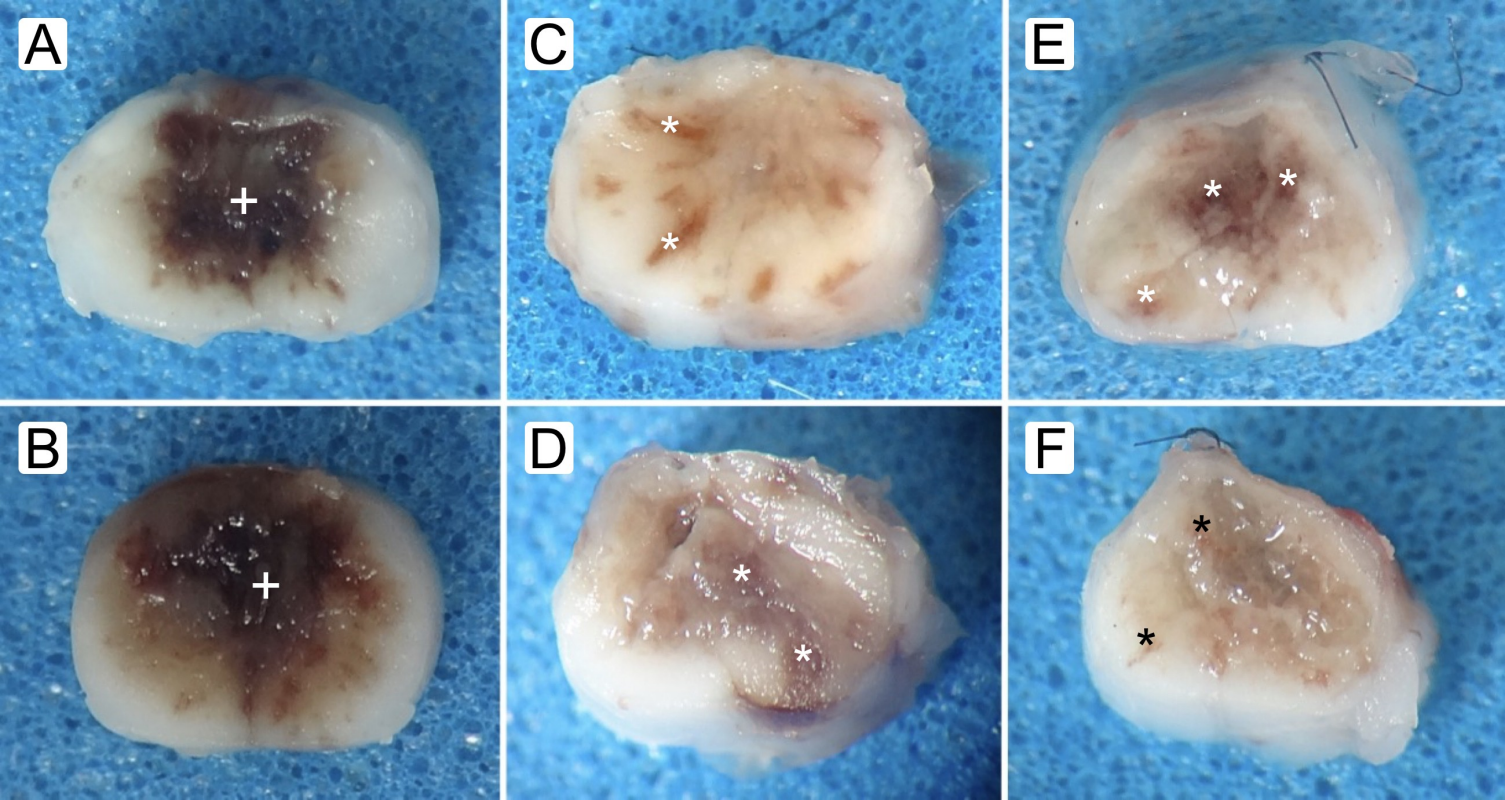
Macroscopic appearance of spaces created by small surgical approach to debridement (SSAD). Representative images of cross sections at epicenter of control (A, B) and SSAD (C-F) specimens. Rats were subjected to both moderate (A, C, and E) and severe (B, D, and F) injuries, and cords were harvested at 1 h (A-D) and 24 h (E, F) after SSAD or control handling. In treated rats (C-F), the spaces created by SSAD appear filled with hydrogel. +, abundance of intramedullary hemorrhagic necrosis (IHN); *, petechial hemorrhaging and scarce IHN.

**Fig 15:**
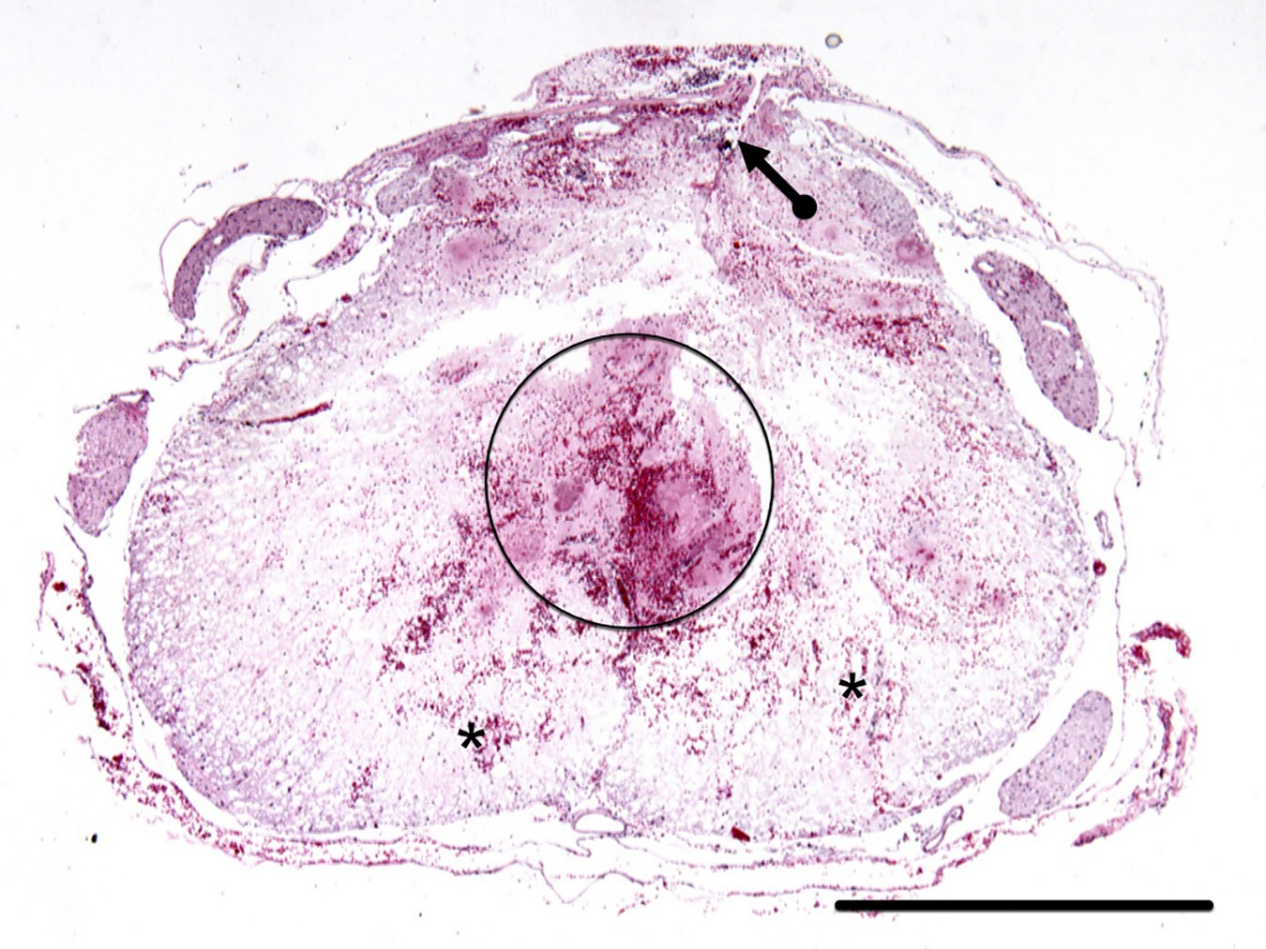
Histologic examination of hydrogel filled intramedullary space created after IHN removal by small surgical approach to debridement (SSAD). Representative image of a transverse section at the epicenter of a specimen from a rat subjected to a SSAD 24 h before euthanasia. The circle shows the hydrogel filled space created by SSAD, partially infiltrated by erythrocytes. The image also shows petechial hemorrhaging (*) in the spared white matter; and the site at which the cord was intervened (arrow). H & E stain. Scale bars: 1 mm.

Macroscopically, specimens of untreated animals showed abundance of IHN, while those from treated rats showed IHN free spaces filled with hydrogel; petechial hemorrhaging and scarce IHN were also observed (Fig 14). Histologically, hydrogel filling the space formed after IHN removal, appeared infiltrated by variable amounts of erythrocytes (Fig 15).

### Discussion

A microsurgical procedure was developed here to create an intramedullary cavity by removing IHN, from both moderately and severely cord injured rats, without further cord damage, for future implantation of regenerative materials in the acute phase after spinal cord contusion in rats.

### Rationale for conducting the present study

Therapies involving cell transplantation and the delivery of restorative bio-molecules to injured cord parenchyma are promising strategies to enhance and complement mechanisms that occur spontaneously after injury and include neuroprotection, axonal regeneration, remyelination, neovascularization, and replacement of damaged cells [34,37–39].

Spinal cord transection and hemisection are the most common injury models used for intralesional implantation interventions since the gap created by the injury itself enables the placement of scaffolds [38]. However, in the clinical setting, the most common type of SCI results from contusions [40–42].

Accordingly, the model we have used here and believe is clinically relevant, was produced by contusion. Actually, it has been shown that cord contusions accurately reproduce most human SCI [40–42].

When looking to make a therapeutic implant into the area of lesion in the acute stage of a spinal cord contusion in rat, there are essentially two problems that must be addressed: in the first place, the spinal cord is so swollen that there is practically no room to place the implant, and secondly, the microenvironment generated by the IHN is potentially detrimental to the implant’s proper functioning. Therefore, we designed the present study addressing both issues: by removing IHN and the risk of its potential toxicity [6,13,14,16,17], we made room in the injured cord tissue for the placement of therapeutic implants.

### Issues related with intervention timing

It is widely accepted that timing of treatment is crucial to achieve the best outcome after SCI; typically, early interventions lead to better results.

Bearing in mind the future clinical application of the procedure developed here, we chose to intervene in the acute phase (one day after injury). Besides, it has been reported that the optimal time window to perform myelotomies is between 8 and 24 h after cord injury [24], and that the timing of maximum hemorrhaging is 12 h after cord contusion in rat [43].

Many preclinical studies using contusion or compression injury models to test the effectiveness of implantation of cells and/or biomolecules, are often delayed one or two weeks after injury, due to the lack of adequate intramedullary space, as well as the hostile nature of the environment in the acutely injured cord [33,39,44,45], hampering the effectiveness of implant protocols in the clinical setting.

### Concerns about the risk of further damage to the injured cord

A key concern in the development of a therapeutic intervention such as the one we describe here is the risk of further damage to spared cord tissue by intraparenchymal manipulation of the injured spinal cord. In fact, microinjections for intralesional delivery of therapeutic materials after acute contusion have been shown to worsen the site of injury [46].

Here, we initially explored early post-intervention outcomes of three paradigms tested by varying the extent of myelotomies. In all cases, IHN removal was successful, although bleeding in the spared cord tissue was excessive following the extensive and medium sized surgical approaches tested (see Figs 1 and 2). Presumably this bleeding resulted from the inevitable rupture of blood vessels, due to the intricate vascular anatomy of the spinal cord. It is reasonable to speculate that blood invading spared tissue will lead to further cord damage. *Per se*, blood infiltrating CNS tissue is toxic [4,6,14,47]. For this reason, the paradigm of SSAD, that does not produce such potentially damaging bleeding, was chosen for all subsequent studies.

Nonetheless, even using the SSAD, undesirable intramedullary and subarachnoid bleeding attributable to the intervention might occur, as observed here in two cases (see Fig 5). This potential complication may cause additional damage to the injured cord tissue, and thereby increase functional impairment. Accordingly, it is advisable to monitor the possible occurrence of this type of bleeding after intramedullary IHN removal. If bleeding does in fact occur, it should be handled immediately to maximize the safety of the intervention and avoid further injury.

### Morphological and functional outcome

Discrete (non-significant) motor improvement of SSAD rats compared to injured controls for both moderate and severe lesions, contrast with reports by others using rats with injury of moderate intensity [24,25]. In fact, our functional results at eight-weeks follow-up, show that the intervention does not produce additional damage.

Unlike minor differences in the amount of spared cord tissue between SSAD and control MI and SI rats, myelination appears to be positively impacted by debridement in both MI and SI rats. It is presumable that SSAD promotes enhanced myelination by providing a suitable environment for oligodendrocyte survival, migration, proliferation and differentiation, a matter beyond the scope of this study.

### Lack of effects of IHN removal on swelling and inflammation

Both edema and acute inflammation have been involved in the pathophysiology of SCI [5,48]. Contrary to our expectations, IHN removal had no detectable effects on cord swelling or acute inflammation as compared to injured only control rats. It is known that after SCI, vasogenic and cytotoxic edema [41,49], as well as acute inflammation [50,51], occur not only at the site of injury but several millimeters cranial and caudal to it. If edema or acute inflammation changes, in fact, did occur at the site of injury after IHN removal, it is possible that they could have gone unnoticed due to the size of the 15-mm long cord tissue specimen, which included not only the site lesion but also the penumbra, or to its tissue composition, which included the dural sac. Like us, others have also observed a lack of enhanced swelling 2 days after cord injury and 24 h after myelotomy [25], compared to the swelling observed in injured only rats, although they did observe differences 4 and 6 days post-injury.

The lack of changes in MPO activity after IHN removal could be attributable to the facts that: 1) MPO is not only expressed in neutrophils but also in other phagocytes (activated microglia and macrophages) at the site of injury and penumbra [52]; 2) IHN was not fully removed; and 3) although most of neutrophils are in necrotic regions after acute SCI [50,51], they can also be found in the subarachnoid space and injured meninges [53].

### Study limitations and future research

In the first series of experiments, independent variables across extensive, medium, and small debridement procedures (i.e. laminectomy, the use of fibrin glue, and the placement of autologous fascia) were considered as a whole for each group and could have contributed, at least in part, to the differences among them.

The injured cord after SSAD was characterized here mainly by histology; besides, to gain an initial perspective on the effects of this procedure on the injured rat as a whole, some functional and analytical studies were performed. Also, a generic hydrogel was placed intralesionally to characterize the appearance and capacity of the intramedullary space created after IHN removal.

However, more in depth animal studies are warranted for a better understanding of how SSAD might modify key areas within the microenvironment of the injury, including molecular events. We believe that the work we have presented here provides a firm basis for future studies aimed at investigating the effectiveness of intralesional implants early after SCI.

### Future clinical implications

In principle, the placement of a restorative implant at the epicenter of a contusive injury requires an appropriate space within which to fit it. The creation of just such an intramedullary cavity, precisely at the site of injury removing IHN (ridding the site of potentially harmful debris) by SSAD, appears reasonable for the placement of therapeutic cells and other regenerative materials in the early stages after cord injury. Studies such as ours provide advanced knowledge crucial for elaborating novel therapeutic approaches for improved management after acute SCI.

### Conclusions

Using a clinically relevant SCI model in the rat, we designed a procedure to create a cavity at the site of injury by removing IHN to eventually house therapeutic implants. Extensive and medium sized surgical approaches to contused cords resulted in unacceptable bleeding in spared cord tissue adjacent to the site of injury, with the added risk of further neural damage. Instead, 24 h after graded contusion, the successful removal of IHN by SSAD gave way to a 10–15 μL intramedullary space. No effects on swelling or acute inflammation was observed in the short run. In the long run, this procedure had minor effect on motor function recovery and on the amount of spared cord tissue, although it did promote limited myelination. Animals general health remained unaffected.
